# Symptom relief of leiomyomatosis peritonealis disseminata with ulipristal acetate

**DOI:** 10.1007/s10397-013-0816-2

**Published:** 2013-11-05

**Authors:** Jasper Verguts, Guy Orye, Sophie Marquette

**Affiliations:** 1 Department of Obstetrics and Gynecology, Jessa Hospital, Stadsomvaart 11, 3500 Hasselt, Belgium; 2 Department of Obstetrics and Gynecology, University Hospital KU Leuven Gasthuisberg, Herestraat 49, 3000 Leuven, Belgium

**Keywords:** Leiomyomatosis peritonealis disseminata, Nodules, Myoma, Ulipristal

## Background

Leiomyomatosis peritonealis disseminata (LPD) are multiple nodules adherent to and superficially invading the peritoneum, mimicking metastatic ovarian carcinoma. Their origin would be metaplastic mesenchymal cells (MC), which in susceptible women MC could originate from surgical myoma fragments [1]. Estrogen may stimulate subcoelomic MC to proliferate, differentiating into myoblasts, myofibroblasts, fibroblasts, and even decidua-like cells [2]. LPD can express sex steroid receptors, hence be promoted by hormonal stimulation, e.g., pregnancy or exogenous intake [3]. LPD is rare (<200 cases reported), essentially benign, though malignant transformation has been reported [4]. LPD often is asymptomatic but can cause abdominal distension.

## Methods

We report the first case of successful management of LPD with ulipristal, a selective progesterone receptor modulator (SPRM), registered for preoperative management of uterine myoma [5, 6]. All procedures followed were in accordance with the ethical standards of the responsible committee on human experimentation (institutional and national) and with the Helsinki Declaration of 1975, as revised in 2000. Informed consent was obtained from the patient for being included in the study as the use of ulipristal in LPD is off-label.

## Findings

**Fig. 1:**
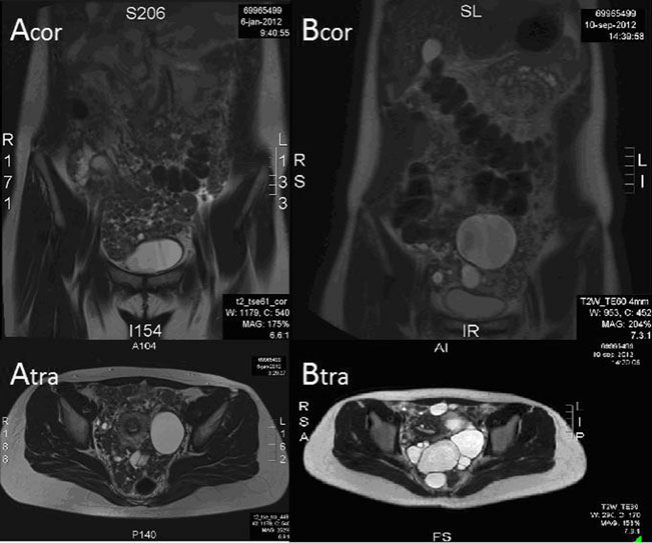
MRI view of LPD. *Acor* coronal view before treatment, *Atra* transverse view before treatment, *Bcor* coronal view after 3 months of treatment, *Btra* transverse view after 3 months of treatment

A 21-year-old nulliparous woman presented with abdominal discomfort coinciding with a pelvic mass stretching beyond the umbilicus. MRI revealed multiple nodules, some with central necrosis, all over the abdomen, apparently not originating from any abdominal organs. She had a hysteroscopic FIGO1 myoma (3-cm diameter) resection 6 months earlier, and 3 years before, a breast fibroadenoma had been removed. Laparotomy disclosed multiple implants over the peritoneal surface and bowels and their meso. Pathology of several biopsies revealed leiomyomatosis, strongly expressing progesterone, but not estrogen receptors. She was first given goserelin acetate 3.6 mg/month (Zoladex®) and tibolone 2.5 mg/day (Livial®) with an objective response on MRI after 3 months. This was continued for 2 years while also on a contraceptive Cu-IUD. The therapy was discontinued because of reassuring images, but she developed menorrhagia and a symptomatic benign ovarian cysts (diameter = 6 cm). Two months of nomegestrol acetate 5 mg/day (Lutenyl®) rested the menorrhagia; however, LPD seemed progressive on MRI (Fig. 1(Acor, Atra)). After obtaining informed consent, she started ulipristal acetate 10 mg/day for 3 months, with response on MRI (Fig. 1(Bcor, Btra)). It was discontinued, assuming that ulipristal would cause long-term suppression [6]. She also developed multiple ovarian cysts up to 12.9 cm, which were punctured because they were symptomatic. After 2 months, she became again symptomatic, coinciding with increase in implant size, so ulipristal was restarted at the same dosage. At this moment, while 12 months on ulipristal, she is asymptomatic. An endometrial biopsy revealed non-atypic hyperplastic proliferative endometrium. A surgical debulking is planned to allow her to stop ulipristal and to conceive.

## Discussion

We believe that this first use of ulipristal for LPD was successful because lesions were expressing progesterone receptors. The subjective and objective responses were striking. Its effect is underscored by a proven reduction in size of lesions and their regrowth following abortion of ulipristal. Though asymptomatic, the disappearance of LPD was incomplete. In addition, she wishes to conceive, so debulking surgery was offered, though the patient reports no side effects, which sharply contrasts with her previous use of GnRH agonists. There is little known on prolonged ulipristal administration. Endometrial atypical hyperplasia may be a concern, though never described for ulipristal. Endometrial biopsy after 12-month exposure is however reassuring. In conclusion, ulipristal acetate may be considered as an adjunct in the management of women with LPD.
